# Native glycosylation and binding of the antidepressant paroxetine in a low-resolution crystal structure of human myeloperoxidase

**DOI:** 10.1107/S2059798322007082

**Published:** 2022-08-09

**Authors:** Lucas Krawczyk, Shubham Semwal, Jalal Soubhye, Salma Lemri Ouadriri, Martin Prévost, Pierre Van Antwerpen, Goedele Roos, Julie Bouckaert

**Affiliations:** a UGSF, CNRS, 50 Avenue de Halley, 59658 Villeneuve d’Ascq, France; b Department of Pharmacognosy, Bioanalysis and Drug Discovery, Faculty of Pharmacy, Université Libre De Bruxelles, Brussels, Belgium; c Structure et Fonction des Membranes Biologiques, Université Libre de Bruxelles, Brussels, Belgium; University of Cambridge, United Kingdom

**Keywords:** human myeloperoxidase, glycosylation, *N*-glycan refinement, thiocyanate, paroxetine

## Abstract

Human myeloperoxidase (MPO) utilizes hydrogen peroxide to oxidize organic compounds and as such plays an essential role in cell-component synthesis, in metabolic and elimination pathways, and in the front-line defence against pathogens. Moreover, MPO is increasingly being reported to play a role in inflammation. The enzymatic activity of MPO has also been shown to depend on its glycosylation. Mammalian MPO crystal structures deposited in the Protein Data Bank (PDB) present only a partial identification of their glycosylation. Here, a newly obtained crystal structure of MPO containing four disulfide-linked dimers and showing an elaborate collection of glycans is reported. These are compared with the glycans identified in proteomics studies and from 18 human MPO structures available in the PDB. The crystal structure also contains bound paroxetine, a blocker of serotonin reuptake that has previously been identified as an irreversible inhibitor of MPO, in the presence of thiocyanate, a physiological substrate of MPO.

## Abstract 2

Myeloperoxidase, prepared from human neutrophil granulocytes, was crystallized in complex with the serotonin-transporter inhibitor paroxetine in crystals containing eight monomers in the asymmetric unit. Each protomer shows up to five asparagine-linked glycan structures. The strategies used and the difficulties encountered in the building and refinement of glycosylation for their improved presentation in the PDB are explained.

## 1. Introduction

The glycosylation of proteins in the PDB has gained attention as the importance of glycosylation becomes better recognized by the scientific community and as a more intense structural characterization and presentation of glycans is being realized (Scherbinina & Toukach, 2020 ▸). Ways to graft carbohydrates as a post-translational modification onto *AlphaFold* prediction models of protein three-dimensional structures (Jumper *et al.*, 2021 ▸) have recently been implemented (Bagdonas *et al.*, 2021 ▸) through the glycan-validation program *Privateer* (Agirre *et al.*, 2015 ▸; Joosten *et al.*, 2022 ▸).

Human myeloperoxidase (MPO) was first isolated in 1941 from purulent pleuritis fluid from tuberculosis patients. When neutrophilic polymorphonuclear leukocytes (neutrophils) entrap microbial or other invasive particulates, they release MPO during degranulation. MPO is a heme Fe^3+^-containing peroxidase with a protoporphyrin IX as the basic structure of its prosthetic group. The porphyrin ring of MPO is covalently attached to the enzyme via two ester bonds (Asp260 and Glu408) and one electron-withdrawing sulfonium linkage (Met409), and features a histidine as a proximal ligand (His502) (Fiedler *et al.*, 2000 ▸).

In the search for a new application for the well known selective serotonin-reuptake inhibitor paroxetine, paroxetine was discovered to have an inhibitory activity on human MPO at nanomolar concentrations (18 n*M*; Soubhye, Aldib *et al.*, 2016 ▸; Soubhye, Chikh Alard *et al.*, 2017 ▸). This opened new opportunities for the treatment of major depressive disorder with inflammatory syndrome (Soubhye, Gelbcke *et al.*, 2017 ▸). Here, we present for the first time the crystal structure (PDB entry 7oih) of paroxetine bound to MPO in the presence of thiocyanate, which hovers above the heme group as previously reported in a crystal structure containing bromide and thiocyanate substrates (Blair-Johnson *et al.*, 2001 ▸). Together with newly performed docking calculations, the crystal structure of the complex provides further insights into the binding and possibly also the inhibition of the peroxidase reaction by MPO.

Mammalian MPO crystal structures have been determined and deposited in the Protein Data Bank (PDB) over the years, and contain one MPO monomer or a disulfide-linked MPO homodimer per asymmetric unit and present partial glycos­ylation. The first human MPO crystal structure was obtained at 1.8 Å resolution, showing multiple halide-binding sites (Fiedler *et al.*, 2000 ▸). Here, we present a new crystal form containing four homodimers of human MPO at 2.6 Å resolution. Using this crystal structure, we obtained an elaborate collection of asparagine-linked glycans at the five *N*-glycosylation sites, Asn323, Asn355, Asn391, Asn498 and Asn729, known from proteomics studies (Van Antwerpen *et al.*, 2010 ▸; Reiding *et al.*, 2019 ▸; Tjondro *et al.*, 2021 ▸). We compared these modifications with those present in the 18 crystal structures (28 monomers) of human MPO in the Protein Data Bank (PDB) and with existing proteomics data. Our MPO crystal structure displays a greater diversity and includes larger *N*-glycans in the electron density than have previously been reported in the PDB, and thereby approaches the analytical results that can be obtained using mass spectrometry. The crystallographic resolution of glycans in crystal structures is helped by ongoing efforts to improve the building and refinement of glycan structures, as described here.

## 2. Materials and methods

### 2.1. Materials

MPO was produced in a pure form (Table 1 ▸), whereas paroxetine hydrochloride was obtained as a lyophilized powder from Sigma–Aldrich–Merck. Highly purified leukocyte MPO (CAS No. 9003-99-0) with a purity index (*A*_430_/*A*_280_) of at least 0.85 was obtained from Planta Natural Products (Kettle & Winterbourn, 1988 ▸).

### 2.2. Crystallization

MPO (10 mg ml^−1^) was mixed with paroxetine inhibitor (25 µ*M*) in 50 m*M* Tris pH 7.4 (Table 2 ▸). Mixtures further reacted with 10 µ*M* hydrogen peroxide also crystallized but as much smaller crystals that did not diffract sufficiently, despite the excess hydrogen peroxide being eliminated before crystallization. It has previously been shown that the activation of MPO with H_2_O_2_ is necessary in order for paroxetine to irreversibly inhibit the enzyme. Irreversible inhibition is potentially due to a covalent linkage, as verified by kinetic studies (Soubhye *et al.*, 2014 ▸), between paroxetine and the active site of MPO. Crystals were only obtained with 0.2 *M* potassium thiocyanate, 0.1 *M* sodium cacodylate, 8%(*w*/*v*) PGA-LM as the precipitant (PGA Screen from Molecular Dimensions).

### 2.3. Data collection and structure resolution

Data were collected on the PROXIMA-1 beamline at Synchrotron SOLEIL, Saint-Aubin, France (Table 3 ▸) and were processed by the automated pipeline using *XDSME* (*XDS Made Easier*; Kabsch, 2010 ▸; Legrand, 2017 ▸). The crystals diffracted to 2.6 Å resolution (Table 3 ▸). PDB entry 4c1m (Forbes *et al.*, 2013 ▸) was used as a model, after removal of the ligand NIH, a trifluoromethyl-substituted aromatic hydroxamate and water molecules, to solve the crystal structure by molecular replacement using *Phaser* (McCoy, 2007 ▸).

### 2.4. Structure refinement of glycosylated MPO

Crystallographic refinement was performed using *phenix.refine* (Afonine *et al.*, 2012 ▸) from the *Phenix* package (Liebschner *et al.*, 2019 ▸) and the refined model was manually adjusted using the graphics program *Coot* (Emsley & Cowtan, 2004 ▸; Emsley & Crispin, 2018 ▸; van Beusekom *et al.*, 2019 ▸) (Table 4 ▸). *MolProbity* was used for protein structure validation (Williams *et al.*, 2018 ▸). Following crystallographic refinement using *Phenix*, the carbohydrate structures of the glycosylations were validated using *Privateer* (Agirre *et al.*, 2015 ▸; Joosten *et al.*, 2022 ▸), which applies Cremer–Pople analysis to determine sugar ring conformations (Cremer & Pople, 1975 ▸). The *Privateer* analysis feeds suggestions for corrections of carbohydrate geometry by rebuilding using *Coot*. Final refinements using *REFMAC*5 (Kovalevskiy *et al.*, 2018 ▸) output an mmCIF that is amenable for PDB deposition and links to other databases such as GlyConnect (Alocci *et al.*, 2019 ▸). This cycle of using *Privateer* for glycan structural valid­ation, *Coot* for model revision and *REFMAC*5 for refinement can be repeated as many times as necessary. Moreover, in *CCP*4 version 8.0 new dictionaries for carbohydrates in the pyranose form have been implemented in the CCP4 Monomer Library, with coordinates reflecting the lowest-energy ring pucker, improved ring torsion restraints and updated geometry (Atanasova *et al.*, 2022 ▸).

### 2.5. Building, refining and validating glycosylation and preparation for deposition in the PDB

The building of glycans has been facilitated in *Coot* using Modules → Carbohydrate, which opens a menu called Glyco that allows the addition of N-linked glycans to the protein and real-space automated refinement in the electron density (Emsley & Crispin, 2018 ▸). At the time of addition, the LINK records are automatically added in the PDB file. If the LINK records between the different carbohydrate residues are missing, or if atoms that leave upon making the glycosidic bonds are not removed, the monosaccharide residues will be pushed apart during refinement due to van der Waals repulsion. Some examples can be found in an excellent overview of how to build and refine glycosylation in protein crystal structures (van Beusekom *et al.*, 2019 ▸). Our general experience was that when handling the 30 glycosylations in the PDB coordinate file of MPO, once the LINK distance surpasses its standard deviation during refinement it will be considered as unlinked, or non­covalently bound, by *phenix.refine* and this will lead to a further separation of the linked glycan. Because the LINK is the only restraint linking the glycan to the protein and may disconnect when the standard deviation is superseded, we set the standard deviation to be large enough in the link.edit file, which is a parameter file containing all nondefault covalent links between residues: 
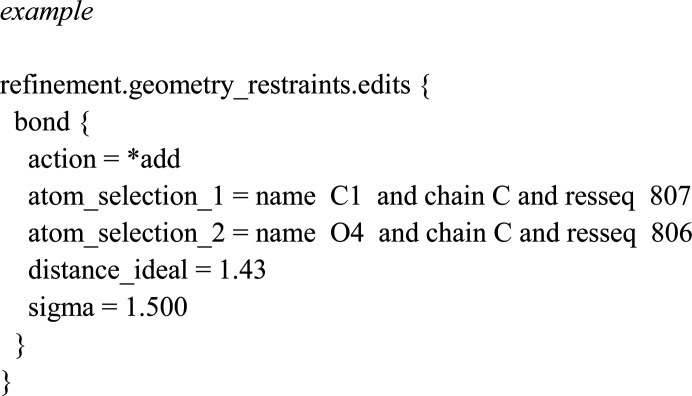


In this example, the sigma is set to be larger than the actual distance to avoid eventual repulsion during refinement. This is especially important in low-resolution structures and with flexible glycan chains where the electron density by itself does not restrain the model sufficiently. Also, stronger restraints are needed within the geometry .cif files of monosaccharides. Therefore, we manually set all of the standard errors on angles, bonds, dihedral angles *etc.* in the implied monosaccharide.cif to half the default value of the CCP4 library of monomer.cif files. Again, this is particularly important at lower resolution, where the data-to-parameter ratio is often too poor to maintain the correct configuration of the monosaccharide, compared with at high resolution (<2.0 Å diffraction resolution).

Deposition of the coordinate file in the PDB needs to take place as a macromolecular Crystallographic Information File (mmCIF). This is equally so for validation prior to PDB deposition. The conversion program for coordinates *pdb_extract* has been integrated into *CCP*4 (Winn *et al.*, 2011 ▸) and the *CCP*4*i* interface (version 5.0 and above). Users can run *pdb_extract* in the *CCP*4 environment. In *pdb_extract*, one defines the polymers as polypeptides given in their one-letter code sequence to output all coordinates inclusive of non­protein atoms into an mmCIF coordinate file. This file can be read in *PyMOL* (Schrödinger), which will display the *N*-glycosidic link to the asparagine as presumed based on a distance that is within the dimensions of a covalent bond. The covalent N-linkage and the glycosidic links between the saccharide units can also be displayed when reading the mmCIF file in the graphics program *ChimeraX* (Pettersen *et al.*, 2021 ▸). Otherwise, the glycan may appear to be disconnected from the protein and broken up into its singular monosaccharide entities.

With the conversion of the coordinate file from .pdb to .cif format for PDB deposition, the glycans will be split off from the protein into separate entities in the event that more than a single *N*-acetylglucosamine is linked to the Asn ND2 atom via a glycosidic N-linkage. For *N*-glycans equal to or extending beyond chitobiose disaccharide [2-acetamido-2-deoxy-β-d-glucopyranose-(1–4)-2-acetamido-2-deoxy-β-d-glucopyranose], 
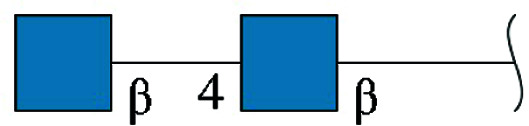


the glycan entities will obtain new chain identities (chain IDs). Within this newly generated chain, the monosaccharide residues will be renumbered starting from 1. These glycan chains receive a chain identity unrelated to the chain identity of the protein monomer that they are glycosylating. It is important that the intramolecular connectivity information remains conserved in the macromolecular model-containing mmCIF upon the assignment of new glycan-chain identities, not least when one has to respond to errors in regard to validation by the PDB. A convenient way to list and decipher all glycosyl­ations in the crystallographic model is to read prosmart-refmac.cif, a model output file from refinement with *REFMAC*5, into the molecular-graphics visualiz­ation program *CCP*4*MG* (McNicholas *et al.*, 2011 ▸). Choosing Glycan viewer from the menu will list the schematic structure of each glycan per protein chain, together with the residue that it glycosylates labelled with its name, number and chain ID (McNicholas & Agirre, 2017 ▸).

### 2.6. Molecular docking of paroxetine for comparison with the crystal structures

The crystal structure of the human MPO structure (PDB entry 7oih) was used as the target structure to perform the docking studies. The crystal structure of one MPO monomer was prepared using the *Protein Preparation Wizard* in the Schrödinger software package (*Protein Preparation Wizard*, *Impact* and *Prime* from release 2017-1; *Epik* from release 2020). Crystallographic water molecules within 5 Å of heteroatoms in the structure were retained and treated as part of the receptor environment.

The initial 3D structure of paroxetine was generated using the *LigPrep* module (Schrödinger release 2017-1). The *Epik* program was used to predict its different protonation states. Docking of paroxetine was carried out in the rigid prepared experimental X-ray structure of MPO using *Glide* (Schrödinger release 2021-1) and was performed in a delimited area (24 × 24 × 24 Å) based on the location of the bound paroxetine in the crystal structure. At most ten docking poses of the ligand were generated and scored using the *Glide* XP function. The docking poses with the highest scores were used for comparison with the crystal structure.

## 3. Results and discussion

### 3.1. Paroxetine binding in the crystal structure of MPO

**Figure 1:**
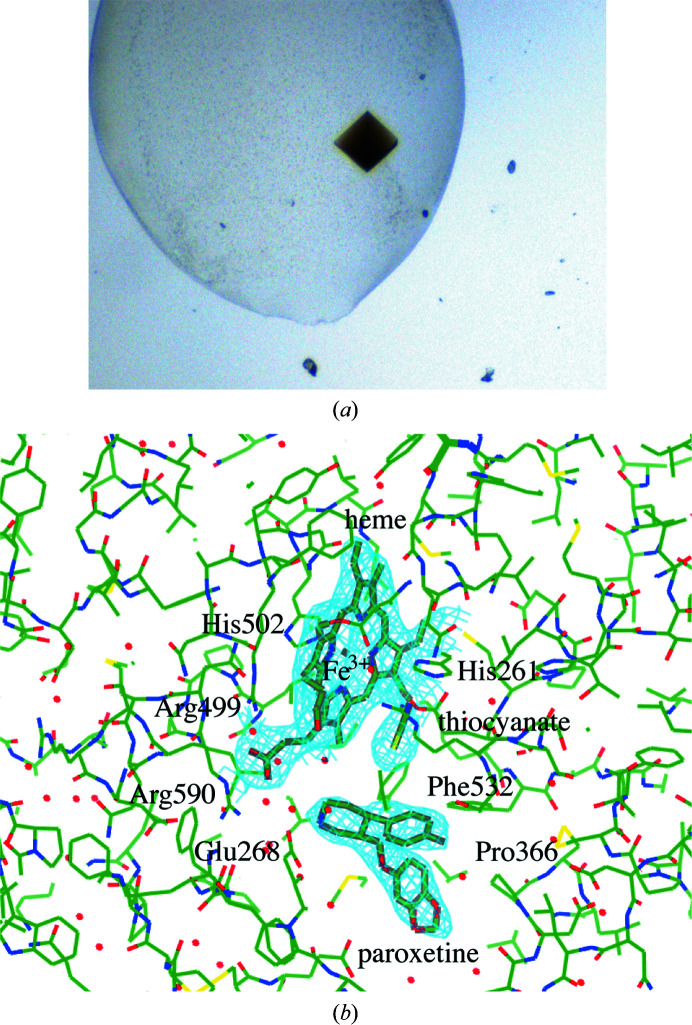
Human MPO crystallized in space group *C*2. (*a*) The greenish colour indicates the presence of Fe^3+^ in the heme. (*b*) Electron density for the heme group of MPO and the nearby binding of thiocyanate and paroxetine, as found in the monomer with chain ID *A*. Amino-acid numbering is based on the pro-MPO crystal structure numbering (Grishkovskaya *et al.*, 2017 ▸), including the signal peptide and pro-peptide. This figure was made using the *CCP*4*MG* molecular-graphics visualization program (version 2.11.0; McNicholas & Agirre, 2017 ▸).

We could capture the binding of the serotonin-transporter inhibitor paroxetine, which is known to be one of the few irreversible MPO inhibitors (Soubhye, Chikh Alard *et al.*, 2017 ▸), in the crystal structure of the native enzyme (Soubhye, Meyer *et al.*, 2016 ▸). Clear electron density is present for paroxetine bound in four of the eight monomers of MPO (Fig. 1 ▸).

**Figure 2:**
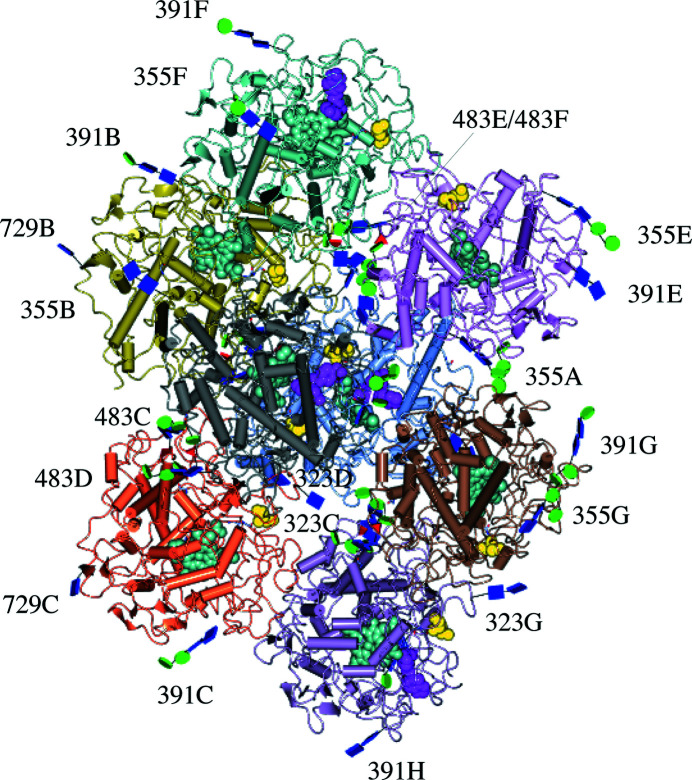
The eight monomers forming four biological assemblies (homodimers *AB*, *CD*, *EF* and *GH*) in the crystal structure of human MPO, with their glycosylation structures. Each chain has a different colour. Monomers *A*, *D*, *F* and *H* have a bound paroxetine inhibitor (magenta), and each catalytic site carries an iron-containing heme group (sea-green) and has an *S*-hydroxy-l-cysteine (yellow) within a distance of 12 Å from the heme group. All *N*-glycosylations start with an *N*-acetylglucosamine (blue square), modifying the labelled asparagine, and many are also further substituted with mannose (green spheres) and fucose (red triangles). This figure was prepared using *CCP*4*MG* (McNicholas & Agirre, 2017 ▸).

The entries to the four other catalytic sites are obstructed by an arginine residue (Arg653) that inserts between the side chain of Asp384, with which it makes a salt bridge, and the carbonyl group of Val576. Every MPO homodimer has only one paroxetine bound and this is with a partial occupancy of paroxetine ranging from 1.0 (full occupancy) in chain *A* to 0.73 (partial occupancy) in chain *H*. The reason for the non­saturation of MPO by paroxetine is probably because of its lower concentration (25 µ*M*) compared with MPO (138 µ*M*) in the crystallization condition. This partial occupancy may have led to the different crystal packing of MPO, with eight monomers in the asymmetric unit, which is unique in the PDB, serendipitously rendering a crystal packing that appears to be favourable for the visualization and determination of the glycosylation of MPO (Fig. 2 ▸).

**Figure 3:**
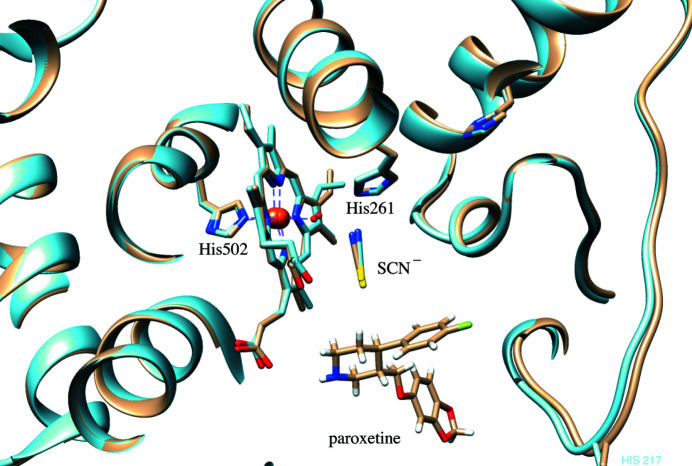
Superposition of PDB entry 1dnu (blue) containing thiocyanate (SCN^−^) (Blair-Johnson *et al.*, 2001 ▸) with chain *A* of the crystal structure of MPO (wheat) bound to SCN^−^ and paroxetine.

Although only one of the monomers of each MPO dimer has paroxetine bound, all eight monomers in the crystal contain thiocyanate (Fig. 1 ▸), which was present at 200 m*M* in the crystallization condition. Thiocyanate, in its negatively charged form SCN^−^, can bind MPO and oxidize to HOSCN, which can induce reversible modifications to mammalian cells that are repairable and thus less damaging (Guo *et al.*, 2020 ▸). In our crystal structure, thiocyanate is found in the same position as reported previously (Blair-Johnson *et al.*, 2001 ▸), hovering above the heme porphyrin ring (Fig. 3 ▸). Its simultaneous presence with paroxetine may have an effect on how paroxetine is positioned in the active site.

**Figure 4:**
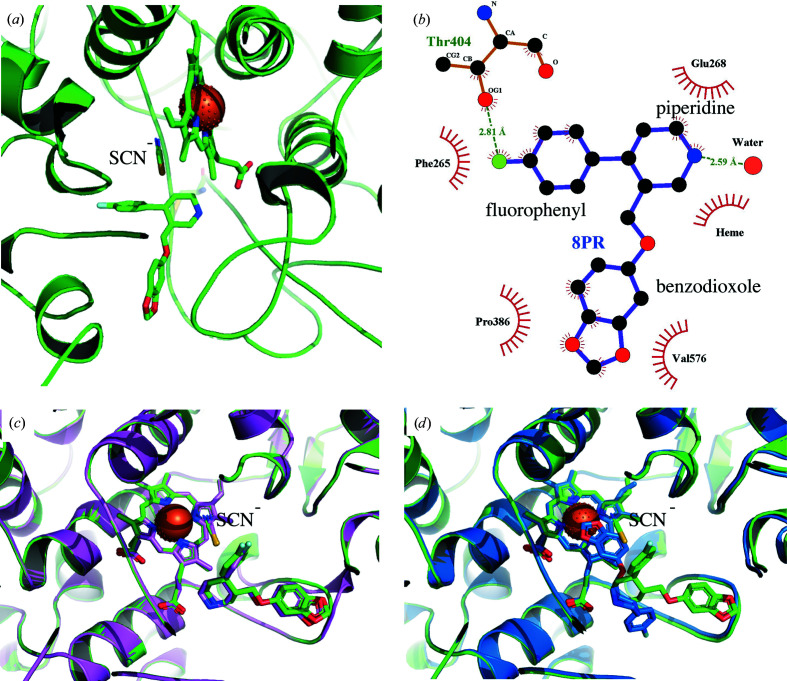
(*a*) MPO crystal structure (green). (*b*) *LigPlot*+ 2D presentation of the paroxetine (PDB ligand ID 8PR) interactions. (*c*) The best pose of paroxetine from the docking including thiocyanate (lilac) superimposed on the crystal structure (green). (*d*) The best pose of paroxetine from the docking without thiocyanate (indigo) superimposed on the crystal structure (green).

In the crystal structure, the benzodioxole group of paroxetine is oriented towards the outside of the active-site cavity and the fluorophenyl group is oriented away from the active-site heme (Fig. 3 ▸). The N atom of the piperidine group is oriented towards the heme group and forms two interactions with the active site: an ionic interaction with a propionate group of the heme and a hydrogen-bond interaction with Glu268 (Figs. 4 ▸ *a* and 4 ▸ *b*).

Globally, the same position and orientation of paroxetine as in the crystal structure are found when SCN^−^ is included in the docking calculations (Fig. 4 ▸ *c*). Without the thiocyanate molecule, paroxetine docks with its benzodioxole group stacked above the heme in place of SCN^−^ (Fig. 4 ▸ *d*). While the interactions of the piperidine group remain conserved, the fluorophenyl and benzodioxole groups flip by about 180°. Therefore, thiocyanate might have caused displacement of the paroxetine, preventing it from entering and anchoring into the active site.

Indeed, in the presented crystal structure paroxetine binds at a position that is different from the position that it needs to adopt for its irreversible interaction with MPO (Fig. 4 ▸). Our structure is however physiologically relevant as thiocyanate is ubiquitous in human plasma and can be elevated by drugs, diet and smoking (van Dalen *et al.*, 1997 ▸; Guo *et al.*, 2020 ▸). Thiocyanate has a much higher (∼730-fold) specificity constant for MPO than chloride, which is considered to be the physiological substrate of MPO (van Dalen *et al.*, 1997 ▸). As such, thiocyanate is likely to be a major substrate of myeloperoxidase in most environments in which this enzyme acts (van Dalen *et al.*, 1997 ▸), making our presented structure relevant to further consideration of paroxetine as an inhibitor of peroxidase activity.

### 3.2. Glycosylation in the crystal structure of MPO

**Figure 5:**
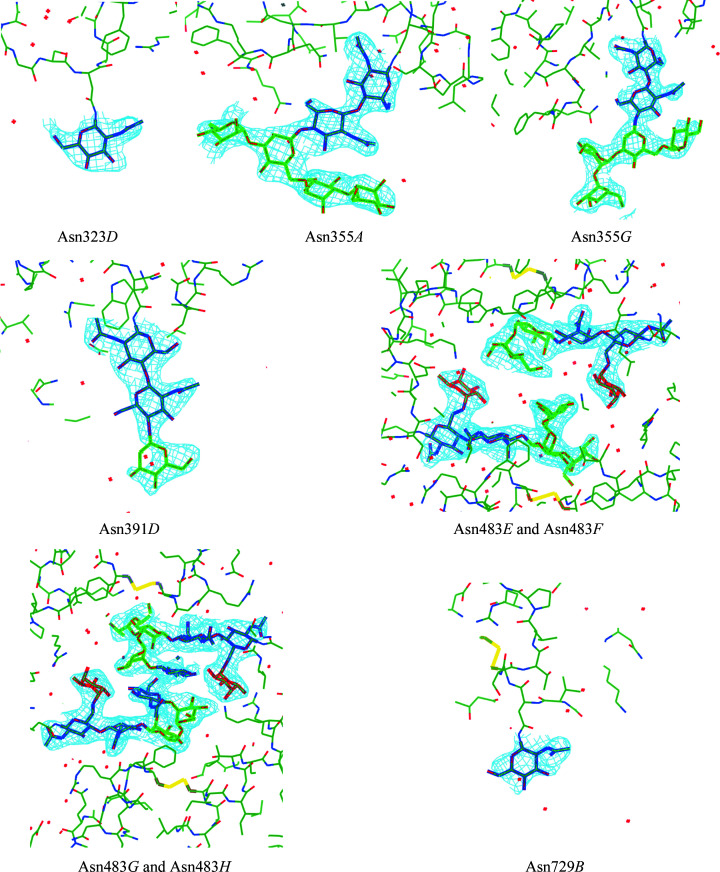
Electron density (blue mesh) defining *N*-glycan structures in the MPO crystals. Some of the glycans are shown, as well as the newly defined FA1[6] glycan on Asn483 of chains *G* and *H*. Mannose is depicted as a green stick model, fucose in red and *N*-acetylglucosamine in blue. This figure was prepared using *CCP*4*MG* (McNicholas & Agirre, 2017 ▸).

Native human MPO has five glycosylation sites identified at positions Asn323, Asn355, Asn391, Asn483 and Asn729 (Van Antwerpen *et al.*, 2010 ▸; Fig. 5 ▸). The structure, activity and regulation of MPO by the natural inhibitor protein ceruloplasmin have been shown to depend on the local *N*-glycosyl­ation pattern (Tjondro *et al.*, 2021 ▸). Microheterogeneity in the site-specific *N*-glycan structures was found to be affected by the localization and maturation status of the enzyme (Reiding *et al.*, 2019 ▸; Ugonotti *et al.*, 2022 ▸; Venkatakrishnan *et al.*, 2020 ▸), with many glycans uniquely identified in mature neutrophils (Tjondro *et al.*, 2021 ▸). Glycosylation of mature MPO was shown to be required for optimal enzymatic activity, possibly through allosteric effects due to interconnectivity, such as between His261 in the distal heme pocket neighbouring the calcium ligand residue Asp262 that is further connected to the Asn355 glycosylation site by an α-helix (Fiedler *et al.*, 2000 ▸).

We determined the (potentially 40) glycan structures on five glycosylation sites per monomer for eight monomers per unit cell in the crystal structure of native human MPO. *Privateer* (Agirre *et al.*, 2015 ▸) was used to validate the glycan structures against the pyranose monomer library from *CCP*4 version 8.0 (Atanasova *et al.*, 2022 ▸). *Privateer* cross-checks the modelled glycans against glycomics databases; in particular, whether the glycan is expected to match an entry in GlyConnect (Alocci *et al.*, 2019 ▸). Ambiguities in the annotation of parts of the glycan structures (motifs) or the whole glycan can be overcome using the GlySTreeM knowledgebase (Daponte *et al.*, 2021 ▸). For example, an α1,4-linked mannose (MAN monomer in the PDB) initially wrongly added as the central mannose in the common trimannose core (M3) was not recognized by GlyConnect until it was corrected to a β1,4-linked central mannose (BMA monomer in the PDB). A very useful feature is that *Privateer* produces interactive 2D graphical plots of the detected glycan trees and the amino acids that they modify (Bagdonas *et al.*, 2020 ▸). Placing the mouse pointer over any of the monosaccharides will display the residue with its name, number and *B* factor from the PDB file.

**Figure 6:**
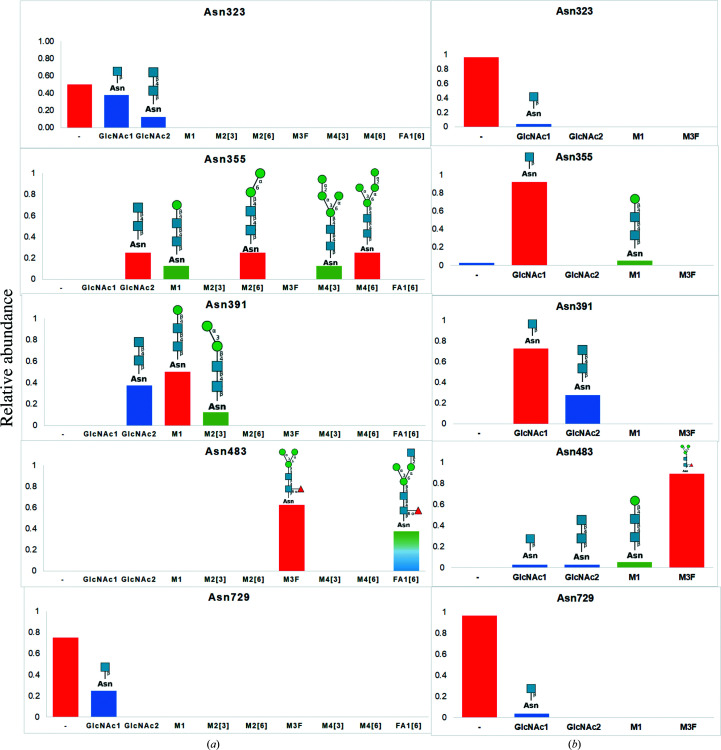
Relative abundance of the *N*-glycans on each glycosylation site as found in the MPO crystal structure (PDB entry 7oih) and comparison with the glycosylation of human MPO structures available from the PDB. (*a*) The repartitioning of the glycans on the eight monomers of our MPO crystal structure, with the predominant glycosylation type(s) highlighted in red. (*b*) The different glycan structures on each glycosylation site for 28 MPO monomers found in the PDB.

The final model contains eight polypeptide chains of mature MPO and 30 glycan chains on an asparagine side-chain ND2 atom, or in brief *N*-glycosylation. No protein *O*-glycosylation was found. The asparagine residues were either predominantly nonglycosylated, or glycosylated with hyper-truncated, paucimannosidic and hybrid *N*-glycans (Fig. 5 ▸), and are also present to some extent in human MPO crystal structures in the PDB (Lütteke *et al.*, 2004 ▸; Fig. 6 ▸).

We compared the glycosylation characterized in our crystal structure with the *N*-glycosylation modelled in the crystal structures of human MPO made available in the PDB (Fig. 6 ▸). Illustrations of protein glycosylation were generated using *DrawGlycan-SNFG* (Cheng *et al.*, 2017 ▸), which applies the Symbol Nomenclature for Glycans (SNFG; Neelamegham *et al.*, 2019 ▸). We can conclude that despite glycosylation in MPO crystal structures receiving little attention, crystallography has the capacity to resolve glycan structures in their diversity and heterogeneity (Fig. 6 ▸ *a* versus Fig. 6 ▸ *b*). This becomes especially true in a space group where a large unit cell permits multiple copies of the same glycoprotein to be present, each of them not subject to the same crystal-packing constraints.

The diversity of glycan structures observed in current MPO crystal structures opens the possibility of comparison with the heterogeneity of these structures present in acquired proteomics data for MPO (Van Antwerpen *et al.*, 2010 ▸; Reiding *et al.*, 2019 ▸; Tjondro *et al.*, 2021 ▸; Table 5 ▸). Reiding and coworkers made a qualitative and quantitative distribution of the glycans after a triplicate LC-MS^2^ run (Reiding *et al.*, 2019 ▸). Isomerisms were not investigated in this study. Tjondro and coworkers obtained the glycoprofiles of MPO secreted by neutrophils by using a reversed-phase LC-ESI-HCD-MS^2^ analysis (Tjondro *et al.*, 2021 ▸). Both studies identified almost the same predominant glycans (PGs), which are M2F on Asn323, M6 on Asn355, M6 on Asn391 and M3F on Asn483. An exception concerns the glycosylation on Asn729. Similar to the large shift from high-mannose and paucimannose glycosylation of Asn355 in the two (biological and validation) MPO batches from Reiding *et al.* (2019 ▸), changes are possible due to the *N*-glycan remodelling that occurs post-biogenesis. *N*-Glycans are more or less susceptible to glycan-processing enzymes depending on the position of the glycosylation site in the structure of the protein that they glycosylate (Mathew *et al.*, 2021 ▸). The glycosylation site may also perform a truly individual protein function, for example only the Asn323 site has the peculiar phosphomannosylation that led Reiding and coworkers to suggest that neutrophils may have repurposed the M6P-mediated trafficking from the lysosomal pathway to populate proteins in their azurophilic granules (Reiding *et al.*, 2019 ▸).

FA1[6] is a hybrid glycan on Asn483 that has not yet been observed in MPO crystal structures but is well represented in the two independent proteomics studies (Table 5 ▸) under the names N3H3F1 (Reiding *et al.*, 2019 ▸) and FA1 (Tjondro *et al.*, 2021 ▸). However, the ‘plus’ of our study is that we can distinguish the isomer because we obtain the three-dimensional structure of the glycan (Fig. 5 ▸). Therefore, we can designate this glycan structure as FA1[6] owing to the presence of *N*-acetylglucosamine on the α1,6-arm of a trimannose *N*-glycan core (Fig. 6 ▸).

**Figure 7:**
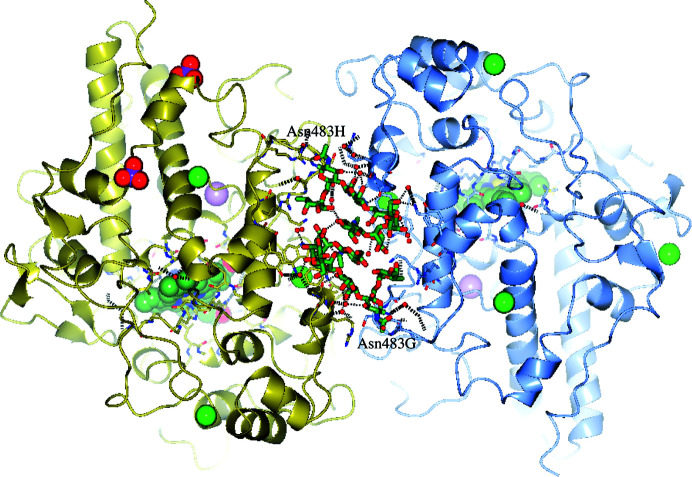
Glycosylation with FA1[6] on Asn483 of both monomer chains *G* (blue) and *H* (gold) of one MPO homodimer. The two *N*-glycans pack tightly with each other at the dimer interface by symmetrically using the *N*-­acetylglucosamine (centre) β1,2-linked to the α1,6-arm of the trimannose core and the fucose α1,6-linked to the core GlcNAc1 (at the two extremities). Intermolecular and intermolecular hydrogen bonds, including water molecules, are shown as black dashed lines. The heme groups are present in both monomers and are shown as sea-green ball-and-stick models. Paroxetine is bound in one monomer per dimer and is not visible here as it is hidden behind the heme group of chain *H*. Green spheres represent chloride ions, pink spheres are calcium ions and red compositions are phosphate ions.

This may seem unusual because during glycan biogenesis the α1,3-arm of the common trimannose core of *N*-glycans is the first one to receive an *N*-acetylglucosamine residue by means of GlcNAc-transferase-1 (Helenius & Aebi, 2001 ▸). It is possible that the observed *N*-acetylglucosamine residue on the α1,6-arm of the trimannose is part of an FA2 structure with both arms, *i.e.* also the α1,3-arm, carrying *N*-acetyl­glucosamine, and that only the GlcNAc on the α1,6-arm is visible in the electron density. This could indeed be the case, as the GlcNAc residue stacks with the noncrystallographic symmetry-related GlcNAc residue of the other monomer of the MPO dimer (centre of Fig. 7 ▸).

The assignment of FA1[6] does not align with isomer assignments for this glycan from previous glycoproteomic research based on the existing knowledge of *N*-glycan biogenesis and in which the retention time of only one of the isomers was known on PGC LC chromatography (Tjondro *et al.*, 2021 ▸). We must stress that FA1[6] may also have been formed from FA2 or higher *N*-glycans by hexosaminidases HexA/B (Ugonotti *et al.*, 2022 ▸). Recently, it has been confirmed that in human neutrophils (F)A2 *N*-glycans formed by the GlcNAc-transferase-1 pathway are retrogradely processed into paucimannosidic glycans. Our finding of FA1[6], carrying an *N*-acetylglucosamine residue, on the α1,6-arm of the trimannose *N*-glycan core may indicate that a truncation of an FA2 *N*-glycan has occurred at the Asn483 glycosylation site. Trace amounts of the precursor of FA1 (Ugonotti *et al.*, 2022 ▸), namely FA2, were indeed found in the earliest proteomics study of MPO (Van Antwerpen *et al.*, 2010 ▸), as well as in a later report (Tjondro *et al.*, 2021 ▸).

These results show us that experimental data by means of crystallography on glycoproteins can not only reveal the partial structures of the glycans that are most well defined by electron density and potentially important for the stability of the protein. It can also inform on the three-dimensional structure of glycosylation and be complementary to results retrieved from glycoproteomic studies that are, in general, limited to the analysis of two-dimensional structures of glycans.

In conclusion, myeloperoxidase prepared from human blood was crystallized in complex with the serotonin-transporter inhibitor paroxetine in crystals containing eight monomers in the asymmetric unit. This renders structural data on a unique set of glycans which until now have not been represented in MPO structures present in the PDB. Each of the five *N*-glycosylation sites is either nonglycosylated or glycosylated with hypertruncated paucimannosidic, short high-mannose and hybrid *N*-glycans, keeping the redox funnel towards the heme group active. The MPO used here was isolated from human neutrophils from healthy donors (Bakkenist *et al.*, 1978 ▸) and its glycosylation had previously been analyzed using mass spectrometry and compared with that of recombinant human MPO (Van Antwerpen *et al.*, 2010 ▸). Its glycans are paucimannose and a dominance of high-mannose glycans. Both studies performing quantitative glycoproteomics (Reiding *et al.*, 2019 ▸; Tjondro *et al.*, 2021 ▸) found exactly the same presence of paucimannose and high mannose as major glycan structures, as they also used human MPO isolated from neutrophil granulocytes from healthy donors. We demonstrate the great potential of crystallographic data to resolve three-dimensional structures, including those of glycans, and explain strategies but also difficulties in the building and refinement of glycosylation for its improved representation in the PDB.

## Supplementary Material

PDB reference: myeloperoxidase, 7oih

## Figures and Tables

**Table 1:** Myeloperoxidase (MPO) production information

Source organism	*Homo sapiens*
Purification grade	Prepared from human blood neutrophils that have been shown by certified tests to be negative for HbsAg and for HCV and HIV antibodies to 98% purity
Purified by	Planta Natural Products, Vienna, Austria
Molecular weight (Da)	Dimer, 145000; monomer, 72500
Complete amino-acid sequence	MGVPFFSSLRCMVDLGPCWAGGLTAEMKLLLALAGLLAILATPQPSEGAAPAVLGEVDTSLVLSSMEEAKQLVDKAYKERRESIKQRLRSGSASPMELLSYFKQPVAATRTAVRAADYLHVALDLLERKLRSLWRRPFNVTDVLTPAQLNVLSKSSGCAYQDVGVTCPEQDKYRTITGMCNNRRSPTLGASNRAFVRWLPAEYEDGFSLPYGWTPGVKRNGFPVALARAVSNEIVRFPTDQLTPDQERSLMFMQWGQLLDHDLDFTPEPAARASFVTGVNCETSCVQQPPCFPLKIPPNDPRIKNQADCIPFFRSCPACPGSNITIRNQINALTSFVDASMVYGSEEPLARNLRNMSNQLGLLAVNQRFQDNGRALLPFDNLHDDPCLLTNRSARIPCFLAGDTRSSEMPELTSMHTLLLREHNRLATELKSLNPRWDGERLYQEARKIVGAMVQIITYRDYLPLVLGPTAMRKYLPTYRSYNDSVDPRIANVFTNAFRYGHTLIQPFMFRLDNRYQPMEPNPRVPLSRVFFASWRVVLEGGIDPILRGLMATPAKLNRQNQIAVDEIRERLFEQVMRIGLDLPALNMQRSRDHGLPGYNAWRRFCGLPQPETVGQLGTVLRNLKLARKLMEQYGTPNNIDIWMGGVSEPLKRKGRVGPLLACIIGTQFRKLRDGDRFWWENEGVFSMQQRQALAQISLPRIICDNTGITTVSKNNIFMSNSYPRDFVNCSTLPALNLASWREAS

**Table 2:** Crystallization

Method	Vapour diffusion
Plate type	Hampton Research, 48-well hanging drop, greased
Temperature (K)	291
Protein concentration (mg ml^−1^)	10
Buffer composition of protein solution	50 m*M* Tris pH 7.4
Composition of reservoir solution	0.2 *M* potassium thiocyanate, 0.1 *M* sodium cacodylate, 8%(*w*/*v*) PGA-LM†
Volume and ratio of drop	2 µl, 1:1 ratio
Volume of reservoir (µl)	100

† Poly-γ-glutamic acid, low molecular weight.

**Table 3:** Data collection and processing Values in parentheses are for the outer shell.

Diffraction source	PX1, Synchrotron SOLEIL
Wavelength (Å)	0.97857
Temperature (K)	100
Detector	PILATUS 6M
Crystal-to-detector distance (mm)	440.5
Rotation range per image (°)	0.1
Total rotation range (°)	180
Exposure time per image (s)	0.1
Space group	*C*2
*a*, *b*, *c* (Å)	155.910, 144.634, 236.454
α, β, γ (°)	90.00, 91.526, 90.00
Mosaicity (°)	0.121
Resolution range (Å)	38.61–2.60 (2.76–2.60)
Total No. of reflections	539003 (83725)
No. of unique reflections	158295 (24289)
Completeness (%)	98.7 (94.5)
Multiplicity	6.44 (6.52)
〈*I*/σ(*I*)〉	7.47 (1.02)†
*R*_meas_	0.195 (1.477)
CC_1/2_ ‡	0.992 (0.426)
Overall *B* factor from Wilson plot (Å^2^)	59.401

† <2.00 from 2.95 Å resolution.

‡ CC_1/2_ (previously called CC_Imean; Evans & Murshudov, 2013 ▸) is the Pearson correlation coefficient obtained by comparing two sets of intensities randomly chosen from the merged crystallographic data. The calculations are usually performed after the two sets of intensities have been divided into thin shells of increasing resolution, so that the dependence of CC_1/2_ on resolution can be determined (Karplus & Diederichs, 2012 ▸; Diederichs, 2016 ▸).

**Table 4:** Structure solution and refinement Values in parentheses are for the outer shell.

Resolution range (Å)	38.96–2.60 (2.67–2.60)
Completeness (%)	98.70 (88.25)
σ Cutoff	None
No. of reflections, working set	156302 (10245)
No. of reflections, test set	1994 (131)
Final *R*_cryst_	0.178 (0.326)
Final *R*_free_	0.219 (0.353)
No. of non-H atoms	
Protein	36629
Ligand	3506
Solvent	2428
Total	41050
R.m.s. deviations	
Bond lengths (Å)	0.011
Angles (°)	1.62
Average *B* factors (Å^2^)	
Overall	58.43
Protein	58.42
Ligand	79.10
Solvent	51.08
Ramachandran plot	
Most favoured (%)	97.34
Allowed (%)	2.42

**Table 5:** MPO glycosylation in the PDB versus proteomics data in the literature The relative abundance of glycans involved in the glycosylation of human MPO in the crystal and the proteome. PG, predominant glycan; N1, GlcNAc1; N2, GlcNAc2; 7oih, the current crystal structure; PDB, 28 MPO monomers from the PDB; RA R, relative abundance in Reiding *et al.* (2019 ▸); RA T, relative abundance in Tjondro *et al.* (2021 ▸); VA, proteomics analysis (nonquantitative) in Van Antwerpen *et al.* (2010 ▸).

	7oih			PDB	Tjondro/Reiding			VA
Glycosylation site	PG	RA T	RA R	PG	PG	RA T	RA R	PG
Asn323	None	0.007	0	None	M2F	0.213	0.192	M4
Asn355	M4	0.044	0.043	N1	M6	0.436	0.475	M6
	M2	0.001	0.002					
	N2	0.001	0					
Asn391	N2	0.001	0	N1	M6	0.314	0.301	M6
Asn483	M3F	0.530	0.357	M3F	M3F	0.530	0.357	M3F
Asn729	None	0.440	0.128	None	None	0.440	0.128	None
					M2F	0.120	0.426	
